# Quantitative Analysis of Sulfur Dioxide Emissions in the Yangtze River Economic Belt from 1997 to 2017, China

**DOI:** 10.3390/ijerph191710770

**Published:** 2022-08-29

**Authors:** Hui Guo, Feng Zhou, Yawen Zhang, Zhen’an Yang

**Affiliations:** 1 Shihezi University, Shihezi 832000, China; 2 Key Laboratory of Southwest China Wildlife Resources Conservation, Ministry of Education, China West Normal University, Nanchong 637009, China; 3 College of Science, Shihezi University, Shihezi 832000, China

**Keywords:** spatial autocorrelation, environmental Kuznets Curve, logarithmic mean divisia index, driving factor, technological innovation

## Abstract

Economic development is responsible for excessive sulfur dioxide (SO_2_) emissions, environmental pressure increases, and human and environmental risks. This study used spatial autocorrelation, the Environmental Kuznets Curve (EKC), and the Logarithmic Mean Divisia Index model to study the spatiotemporal variation characteristics and influencing factors of SO_2_ emissions in the Yangtze River Economic Belt (YREB) from 1997 to 2017. Our results show that the total SO_2_ emissions in the YREB rose from 513.14 × 10^4^ t to 974.00 × 10^4^ t before dropping to 321.97 × 10^4^ t. The SO_2_ emissions from 11 provinces first increased and then decreased, each with different turning points. For example, the emission trends changed in Yunnan in 2011 and in Anhui in 2015, while the other nine provinces saw their emission trends change during 2005–2006. Furthermore, the SO_2_ emissions in the YREB showed a significant agglomeration phenomenon, with a Moran index of approximately 0.233–0.987. Moreover, the EKC of SO_2_ emissions and per capita GDP in the YREB was N-shaped. The EKCs of eight of the 11 provinces were N-shaped (Shanghai, Zhejiang, Anhui, Jiangxi, Sichuan, Guizhou, Hunan, and Chongqing) and those of the other three were inverted U-shaped (Jiangsu, Yunnan, and Hubei). Thus, economic development can both promote and inhibit the emission of SO_2_. Finally, during the study period, the technical effect (approximately −1387.97 × 10^4^–130.24 × 10^4^ t) contributed the most, followed by the economic (approximately 27.81 × 10^4^–1255.59 × 10^4^ t), structural (approximately −56.45 × 10^4^–343.90 × 10^4^ t), and population effects (approximately 4.25 × 10^4^–39.70 × 10^4^ t). Technology was the dominant factor in SO_2_ emissions reduction, while economic growth played a major role in promoting SO_2_ emissions. Therefore, to promote SO_2_ emission reduction, technological innovations and advances should be the primary point of focus.

## 1. Introduction

Sulfur dioxide (SO_2_) is a major atmospheric pollutant. The total global emissions (excluding volcanic eruptions) in 2014 was estimated to have been 105.4 Tg (interquartile range of 95.8–119.8 Tg) [1], and they were primarily derived from the combustion of sulfur containing minerals [2]. With rapid urbanization and industrialization, energy sources are being consumed at a substantial rate, which increases SO_2_ emissions [3]. High SO_2_ emissions can have significant impacts on human health, climate, economies, and ecosystems [1]. For example, SO_2_ contamination increases the risk of preterm birth [4], chronic obstructive pulmonary disease (COPD) [5], and acute respiratory disease [6]. A previous study found that, for every 10 µg/m^3^ increase in SO_2_, the risk of acute myocardial infarction and COPD-related hospital admissions in Iran increased by 2.7% and 2.0%, respectively [5]. Furthermore, in 2015, 3 million people died from COPD worldwide [7]. Additionally, SO_2_ pollution can cause acid rain, as witnessed in 1995, when SO_2_-induced acid rain resulted in more than 110 billion yuan in damages, equivalent to 2% of the GDP [8]. Moreover, high atmospheric SO_2_ concentrations can be detrimental to plant growth and can damage ecosystem stability [9]. Overall, SO_2_ pollution can cause substantial socioeconomic losses, and thus is an obstacle to the sustainable economic development of various countries [10] and a major public health issue in developing countries [11]. Substantial previous research has been conducted to investigate ways to mitigate SO_2_ pollution and achieve more sustainable development options, including adopting various methods to study SO_2_ emissions (Table 1). For example, the spatial autocorrelation method has been used to test the spatial correlation of SO_2_ emissions [9,12], and the Environmental Kuznets Curve (EKC) has been used to analyze the relationship between economic growth and SO_2_ emissions [13]. The spatial autocorrelation method incorporates both global and local spatial autocorrelation, allowing the spatial distribution characteristics of research subjects to be analyzed, as well as the heterogeneous characteristics of any spatial distribution differences [12]. For example, previous studies have used this method to study the agglomeration characteristics of pollutants such as wastewater [14], carbon dioxide (CO_2_) [15], fine particulate matter (PM_2.5_) [16], and SO_2_ [12], and have shown that there is a positive spatial autocorrelation of SO_2_ concentration in China; that is, the SO_2_ concentration in an area will increase because air pollutant spread is affected by nearby areas [9,12]. The EKC has been used to explain the relationship between pollutants and economic development, such as CO_2_ [17], SO_2_ [13], nitrous oxide (NO_2_) [18], and wastewater [14]. According to the EKC, the relationship between national incomes and their contribution to environmental degradation resembles an inverted U-shaped curve [19]. This means that in the early stages of economic development, the increase of economic activities and the structural changes from agricultural to industrial production often lead to an intensification of environmental pollution [20]. After a certain income level is achieved, environmental pollution is reduced through the implementation of cleaner technologies, stricter environmental policies, and structural changes towards light industry and service sectors [20]. However, numerous studies show that, in addition to an inverted U shape, the EKC can also have U, N, inverted N, monotonic increasing, and monotonic decreasing shapes [13,14,21]. Although spatial autocorrelation and the EKC can explore the characteristics of pollutant emission agglomeration and its relationship with economic development, they cannot explain the reasons behind these characteristics and relationships [22]. Researchers have used various other methods to further analyze the factors affecting SO_2_ emissions (Table 1). For example, the stochastic impacts by regression on population, affluence, and technology (STIRPAT) model was used to study the influence of the dominant SO_2_ emission factors [3,23]. Additionally, the Logarithmic Mean Divisia Index (LMDI) and structural decomposition analysis (SDA) have been used to study the effects of direct and indirect factors on SO_2_ emissions [11,24,25,26]. These analyses showed that technological progress, industrial structure, economic growth, and population factors have obvious effects on SO_2_ emissions; technological progress and population factors generally reduce SO_2_ emissions [11,26], while economic growth increases the use of fossil fuels, promoting SO_2_ emissions [10,11,13]. Owing to the accuracy of LMDI decomposition, its lack of unexplained residuals, and its high adaptability [14], LMDI was used in this study to analyze the effects of technical factors, industrial structure, population factors and economic factors on SO_2_ emissions. The aforementioned studies on SO_2_ emissions mostly focus on EKC analysis or the driving factors of SO_2_ emissions, but they rarely combine these two approaches. Therefore, this study first analyzed the changes in SO_2_ emissions during the study period, then analyzed the agglomeration characteristics of SO_2_ emissions and emission changes with economic using spatial autocorrelation and EKC-analysis, and finally analyzed the driving factors using LMDI, so as to achieve a comprehensive analysis of SO_2_ emissions and make reasonable policy suggestions.

Rapid urbanization and industrialization led China to become the largest global energy consumer in 2010 [8]. The dominant energy source in China is coal, which causes substantial SO_2_ emissions and makes China a major global SO_2_ emitter [27]. This abundance of SO_2_ emissions has damaged socioeconomic sectors in China and its neighboring countries, including Japan and South Korea [28]. While reducing emissions is an important domestic and international goal, the economic development of China is inseparable from its energy consumption, making it vital to find a solution that can reduce SO_2_ emissions without affecting economic growth [29]. The Yangtze River Economic Belt (YREB) spans the eastern, central, and western regions of China. The region is home to nearly 45% of China’s population and approximately 20% of the country’s land area and accounts for 50% of its total economic output [30]. It is the economic belt with the greatest economic strength and the most important strategic position [31], making it an important region for China to achieve its 2030 sustainable development goals [30]. The YREB is the inland river economic belt with the largest population and the most complete urban system and global influence in the world [30,31]. It has complex and varied topography and diverse ecosystem structures in its upper and lower reaches. Different regions of the YREB have different basic conditions and levels of economic and social development. The downstream regions (Shanghai, Jiangsu, Zhejiang and Anhui) have a good development foundation and have entered the mature stage of urban agglomeration development; the upstream regions (Chongqing, Sichuan, Guizhou and Yunnan) are still in the initial stage of regional development; and the middle reaches have experienced rapid urbanization and are now advancing to a higher level of development [32].Therefore, SO_2_ emission studies in the YREB and its provinces can serve as references of China’s implementation of regional coordinated development strategies, sustainable development, and SO_2_ emission reduction for other developing countries.

This study used the spatial autocorrelation, EKC, and LMDI methods to study the spatial characteristics and driving factors of SO_2_ emissions in the YREB. Moreover, the time range of previous studies rarely exceeded 20 years (Table 1); therefore, to better analyze SO_2_ emission changes, we set the study time range as 1997–2017. The objectives of this study were to analyze SO_2_ emission characteristics of the YREB and its 11 provinces from 1997 to 2017, study the impact of four driving factors (technological effect, industrial structure, economic effect, and population effect) on SO_2_ emissions in the YREB and its 11 provinces, and put forth specific suggestions for SO_2_ pollution control.

## 2. Materials and Methods

### 2.1. Data Sources

**Figure 1:**
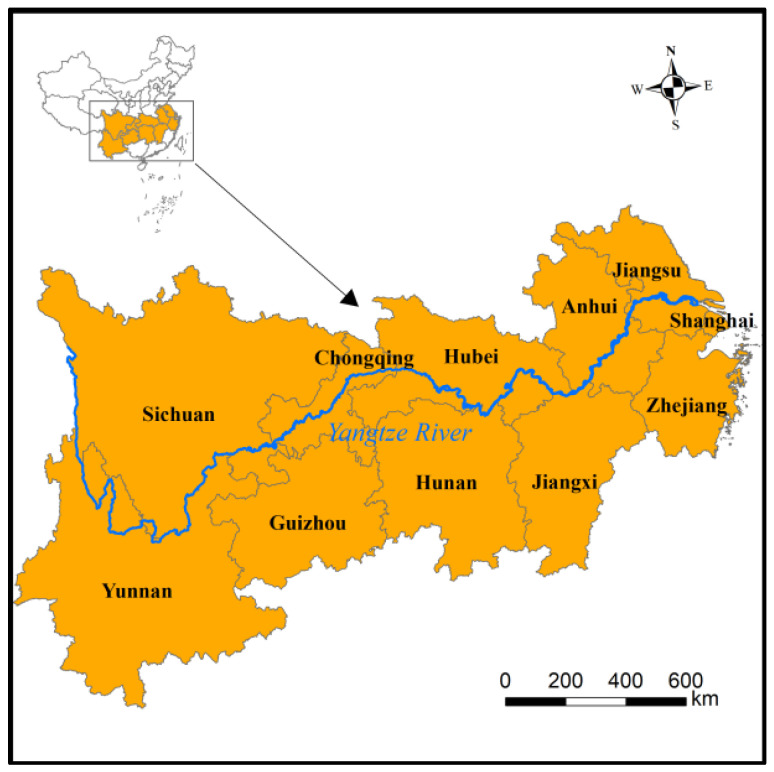
Location of the Yangtze River Economic Belt.

In this study, the YREB and its 11 provinces, namely Shanghai, Jiangsu, Zhejiang, Anhui, Jiangxi, Hubei, Hunan, Chongqing, Sichuan, Yunnan, and Guizhou, were selected as the research areas (Figure 1). The study time range was 1997–2017 and data from all 11 provinces were used. The total SO_2_ emissions, GDP, population number, and industrial added value of the 11 provinces were all collected from the China Statistical Yearbook from 1997–2017. The per capita GDP of the 11 provinces was calculated by dividing the GDP by the total population.

### 2.2. Global Spatial Autocorrelation

Spatial autocorrelation analysis is based on the first law of geography, and can be used to quantitatively measure the degree of interdependence the same variable in different spatial locations, and determine the spatial correlation and spatial heterogeneity of attribute values in different regions [12,14,33]. Previous studies have shown that SO_2_ emissions have strong cross-regional and agglomeration characteristics; therefore, this method can be used to explore the spatial agglomeration characteristics of SO_2_ emissions [9,12]. In this study global Moran’s *I* was used to explore the spatial correlation of SO_2_ emissions as follows [14]:

(1) $$I=\frac{n\sum_{i}\sum_{j}W_{\mathrm{ij}}\left(X_{i}-\bar{X}\right)\left(X_{j}-\bar{X}\right)}{(\sum_{i}\sum_{j}W_{\mathrm{ij}})\sum_{i}{\left(X_{i}-\bar{X}\right)}^{2}}$$

where n is the number of study subjects, X_i_ represents the total SO_2_ emissions in province i, X_j_ represents the total SO_2_ emissions in province j, and W_ij_ represents the spatial weights of elements i and j. The value of global Moran’s *I* range from [−1, 1], where the sign represents the correlation type and the absolute value represents the intensity. When *I* > 0, there is a positive correlation and the closer *I* is to 1, the more spatial agglomeration characteristics the SO_2_ emissions have; when *I* < 0, there is a negative correlation, and the closer *I* is to −1, the more spatially discrete characteristics the SO_2_ emissions have; finally, when *I* = 0, there is no correlation between the SO_2_ emissions in different regions [9,12,14,33]. Moreover, the correlation of this index was evaluated via the *Z* score (standard deviation) and *p* value (i.e., when *Z* < −1.96 or *Z* > + 1.96 and *p* < 0.05, the SO_2_ emissions had significant spatial autocorrelation) [14].

### 2.3. Environmental Kuznets Curve

The EKC has been widely used to study the link between environmental pollution and economic growth. Previous research has shown that the EKC has six types, the inverted U, U, N, inverted N, monotonic increasing, and monotonic decreasing curves [13,14,21]. This means that, with the improvement of income level, environmental quality can show a downward (inverted U, inverted N, monotonic decreasing) or upward trend (U, N, monotonic increasing). This study analyzed the relationship between SO_2_ pollution and economic development during 1997–2017 in the YREB using Statistical Products and Services Solution 19.0 software (International Business Machines Corp., Armonk, NY, USA), as follows [22]:

(2) $$Y_{\mathrm{it}}=a+b_{1}X_{\mathrm{it}}+\varepsilon$$

(3) $$Y_{\mathrm{it}}=a+b_{1}X_{\mathrm{it}}+b_{2}X_{\mathrm{it}}^{2}+\varepsilon$$

(4) $$Y_{\mathrm{it}}=a+b_{1}X_{\mathrm{it}}+b_{2}X_{\mathrm{it}}^{2}+b_{3}X_{\mathrm{it}}^{3}+\varepsilon$$

where Y_it_ represents the SO_2_ emission of Province i in year t; a is the intercept term; b_1_, b_2_, and b_3_ are the coefficients of the primary term, quadratic term, and cubic term respectively; X_it_ represents the GDP of Province i in year t; and ε represents the random error term.

### 2.4. Logarithmic Mean Divisia Index 

In this study, the LMDI model was used to analyze change of SO_2_ emissions in the YREB between 1997 and 2017 in response to four driving factors: technology effect, industrial structure, economic effect, and population effect. According to the LMDI model, SO_2_ emission changes within a certain period can be expressed as [14]:

(5) $$W^{t}=\sum_{i}^{n}W_{i}^{t}=\sum_{i}^{n}\frac{W_{i}}{V_{i}}\cdot \frac{V_{i}}{G_{i}}\cdot \frac{G_{i}}{P_{i}}\cdot P_{i}=\sum_{i}^{n}W_{\mathrm{tec},i}\cdot W_{\mathrm{str},i}\cdot W_{\mathrm{eco},i}\cdot W_{\mathrm{pop},i}$$

where W^t^ represents the total SO_2_ emissions in year t; W^t^_i_ is the total SO_2_ emission in year t and region i; W_i_ is the SO_2_ emissions in region i; V_i_ is the industrial added value in region i; G_i_ is the GDP in region i; P_i_ is the total population in region i; n is the number of research areas; W_tec,i_ = W_i_/V_i_, is the technical effect of region i; W_str,i_ = V_i_/G_i_, is the industrial structure effect of region i, reflecting the impact of industrial structure changes on SO_2_ emissions; W_eco,i_ = G_i_/P_i_, is the economic effect of region i, indicating the impact of regional economic development on SO_2_ emissions; W_pop,i_ is the population size effect of region i; and ∆W_tec,i_, ∆W_str,i_, ∆W_eco,i_, and ∆W_pop,i_, represent the contribution value of the technology effect, industrial structure effect, economic development effect, and population effect to the SO_2_ emissions in region i, respectively. A contribution value greater than 0 indicates that the effect promotes SO_2_ emissions, and an opposite contribution value less than 0 indicates that the effect suppresses SO_2_ emissions. The decomposition formula is as follows [14]:

(6) $$\Delta W_{\mathrm{tec},i}=\frac{W_{i}^{t}-W_{i}^{0}}{\ln W_{i}^{t}-\ln W_{i}^{0}}\cdot \ln\;\left(\frac{W_{\mathrm{tec},i}^{t}}{W_{\mathrm{tec},i}^{0}}\right)$$

(7) $$\Delta W_{\mathrm{str},i}=\frac{W_{i}^{t}-W_{i}^{0}}{\ln W_{i}^{t}-\ln W_{i}^{0}}\cdot \ln\;\left(\frac{W_{\mathrm{str},i}^{t}}{W_{\mathrm{str},i}^{0}}\right)$$

(8) $$\Delta W_{\mathrm{eco},i}=\frac{W_{i}^{t}-W_{i}^{0}}{\ln W_{i}^{t}-\ln W_{i}^{0}}\cdot \ln\;\left(\frac{W_{\mathrm{eco},i}^{t}}{W_{\mathrm{eco},i}^{0}}\right)$$

(9) $$\Delta W_{\mathrm{pop},i}=\frac{W_{i}^{t}-W_{i}^{0}}{\ln W_{i}^{t}-\ln W_{i}^{0}}\cdot \ln\;\left(\frac{W_{\mathrm{pop},i}^{t}}{W_{\mathrm{pop},i}^{0}}\right)$$

where W^0^_i_ represents the emission of SO_2_ in the base year; W^t^_tec__,i_ represents the technical effect of region i in year t; W^t^_str__,i_ represents the industrial structure effect of region i in year t; W^t^_eco__,i_ represents the economic effect of region i in year t; and W^t^_pop__,i_ represents the population effect of region i in year t.

## 3. Results

### 3.1. Spatiotemporal Characteristics of the SO_2_ Changes

**Figure 2:**
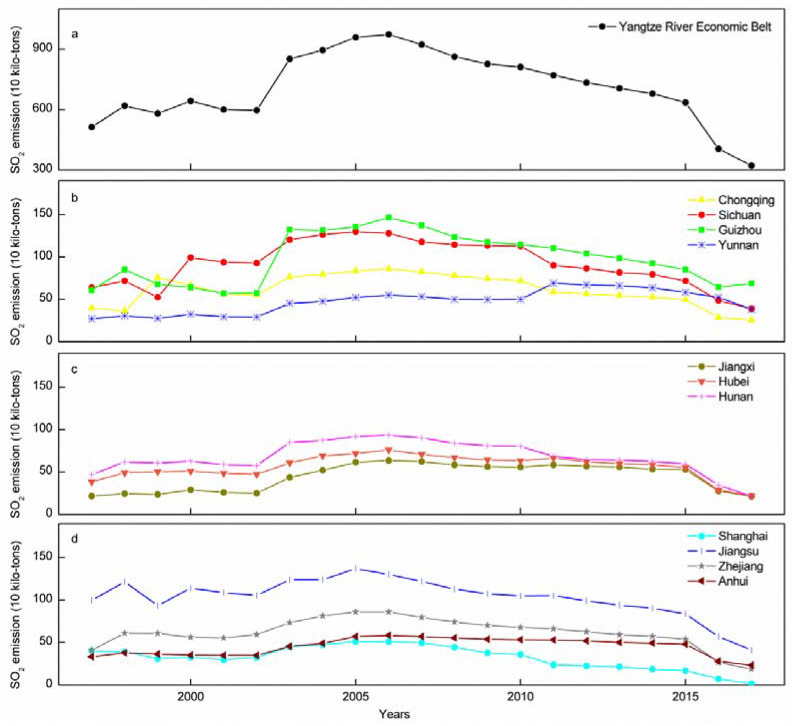
SO_2_ emission changes in Yangtze River Economic Belt (**a**) and different provinces (Chongqing, Sichuan, Guizhou and Yunnan, (**b**); Jiangxi, Hubei and Hunan, (**c**); Shanghai, Jiangsu, Zhejiang and Anhui, (**d**)) during 1997–2017.

The total SO_2_ emissions along the YREB increased from 513.14 × 104 t in 1997 to 974.00 × 104 t in 2006 before dropping to 321.97 × 104 t in 2017 (Figure 2a). The SO_2_ emission trends in Guizhou, Chongqing, Sichuan, Jiangxi, Hubei, Hunan, Shanghai, Jiangsu, and Zhejiang were similar to those in the YREB, that is, the SO_2_ emissions first increased and then decreased, reaching their peak in 2005–2006 (Figure 2b–d). The SO_2_ emissions in Yunnan increased from 1997 to 2011 before decreasing from 2011 to 2017 (Figure 2b), and the SO_2_ emissions in Anhui increased from 1997 to 2005, remained stable from 2005 to 2015, and decreased from 2015 to 2017 (Figure 2d). The SO_2_ emissions of the 11 provinces in the YREB all rose sharply in 2002 and declined drastically in 2015 (Figure 2b–d). Jiangsu showed the highest average emissions (103.45 × 10^4^ t), followed by Guizhou, Sichuan, Hunan, Zhejiang, Chongqing, Hubei, Yunnan, Anhui, and Jiangxi (approximately 44.21 × 10^4^–97.96 × 10^4^ t), while Shanghai showed the lowest (32.45 × 10^4^ t) (Figure S1).

### 3.2. Global Spatial Autocorrelation Analysis of SO_2_ Emissions

From 1997 to 2017, the global Moran’s *I* values of the regional SO_2_ emissions of the YREB were all greater than 0, and passed the significance test (*p* < 0.05), showing a trend of first increasing and then decreasing. Specifically, it increased from 0.308 in 1997 to 0.987 in 2005 and then decreased to 0.233 in 2015 (Table 2).

### 3.3. EKC Study of SO_2_ Emissions

**Figure 3:**
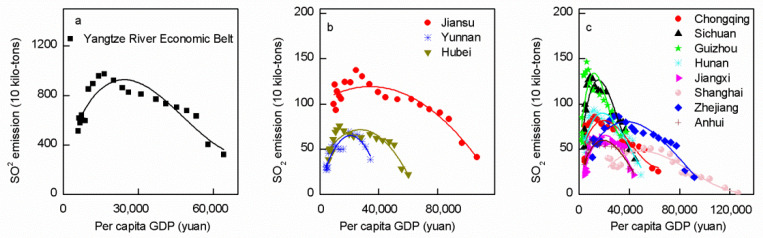
Classification of EKC of Yangtze River Economic Belt (**a**) and different province (inverted U type, (**b**); N type, (**c**)) during 1997–2017.

During 1997–2017, the EKC of the total SO_2_ emissions and per capita GDP in the YREB was N-shaped (Figure 3a, Table S1), the EKCs of Jiangsu, Yunnan, and Hubei were inverted U-shaped (Figure 3b, Table S1), and the EKCs of Shanghai, Zhejiang, Anhui, Jiangxi, Sichuan, Guizhou, Hunan, and Chongqing were N-shaped (Figure 3c, Table S1).

### 3.4. Driving Factors of SO_2_ Emissions 

The contribution value of technology in the YREB was greater than 0 from 1998–2000 and less than 0 from 2001–2017. It decreased from 130.24 × 10^4^ t in 1998 to −1387.97 × 10^4^ t in 2014, and then increased to −1330.47 × 10^4^ t by 2017 (Table 3). The contribution value of the structural effect was less than 0 in 1998–2000 and greater than 0 in 2001–2017. It increased from −56.45 × 10^4^ t in 1998 to 343.90 × 10^4^ t in 2010, and then decreased to 129.03 × 10^4^ t by 2017 (Table 3). The contribution value of the economic and population effects were greater than 0 from 1998–2017, showing increasing trends followed by decreasing trends. The contribution value of the economic effect reached its maximum value of 1255.59 × 10^4^ t in 2014, while the contribution value of the population effect reached its maximum value of 39.70 × 104 t in 2015 (Table 3). Among the four driving factors, the technical effect contributed the most (approximately −1387.97 × 10^4^–130.24 × 10^4^ t), followed by the economic effect (approximately 27.81 × 10^4^–1255.59 × 10^4^ t), the structural effect (approximately −56.45 × 10^4^–343.90 × 10^4^ t), and then the population effect (approximately 4.25 × 10^4^–39.70 × 10^4^ t) (Table 3). 

The average contribution value of the technological effect in the 11 provinces ranged from −161.33 × 10^4^ t to −24.08 × 10^4^ t, primarily showing negative effects (Table 3). The average contribution value of the economic effect in the 11 provinces ranged from 32.07 × 10^4^–124.50 × 10^4^ t, showing a primarily positive influence. In 10 of the provinces, the structure effect was predominantly positive (approximate average contribution value: 1.10 × 10^4^–34.30 × 10^4^ t), while its average contribution value in Yunnan was −1.47 × 10^4^ t (Table S2). The population effect was positive (0.15×10^4^–9.30 × 10^4^ t) in eight of the provinces, but negative in Sichuan (−1.18 × 10^4^ t), Hubei (−0.29 × 10^4^ t), and Chongqing (−1.16 × 10^4^ t) (Table S2). These results show that the technological effect contributed the most to SO_2_ emissions in the 11 provinces, followed by the economic and structural effects, while the population effect contributed the least (Table S2).

## 4. Discussion

### 4.1. Spatiotemporal Variation of SO_2_ Emissions

Total SO_2_ emissions in the YREB fell from 513.14 × 10^4^ t in 1997 to 321.97 × 10^4^ t in 2017 (Figure 2a), showing that the SO_2_ pollution situation in the YREB is gradually improving. This decline may be owing to the implementation of emission policy and emission reduction measures [9]. For example, in December 2007, the State Council issued the “Eleventh Five-Year Plan for National Environmental Protection”, which required SO_2_ emissions in 2010 to be reduced by 10% compared with those from 2005 [34]. Furthermore, in 2013, the Air Pollution Prevention and Control Action Plan was released [35] and the Twelfth Five-Year Plan proposed a target of further reducing SO_2_ emissions by 10% by the end of 2015 compared with those from 2010 [36]. To achieve these goals, a series of SO_2_ emissions reduction measures have been adopted in various YREB regions. For example, in 2008, Jiangsu increased the desulfurization facilities of coal-fired units by 6.59 million kilowatts and closed and eliminated 4326 “small chemical” production enterprises [37], while Sichuan implemented total coal consumption control in 14 cities and eliminated 120,000 old cars in 2013 [38]. SO_2_ emissions from the YREB and its 11 provinces rose sharply in 2002, potentially resulting from the economic growth acceleration that China experienced after 2002, which increased energy-intensive and polluting industry investments and caused massive energy consumption, boosting SO_2_ emissions. For example, the GDP growth rate of the YREB rose from 11% in 2002 to 16% in 2003, and the growth rate of national energy consumption rose from 9% in 2002 to 16% in 2003 [39]. SO_2_ emissions from the YREB and its 11 provinces plummeted in 2015 (Figure 2), likely owing to the Prevention and Control of Atmospheric Pollution Law that came into force on 1 January 2016, which strengthened supervision and made specific provisions on prominent issues in the prevention and control of SO_2_ pollution, such as prohibiting the import, sale, and burning of coal that does not meet quality standards and encouraging the burning of high-quality coal [40].

Emission decline began in Yunnan in 2011 and Anhui in 2015, while that of the other nine provinces began during 2005–2006 (Figure 2), showing that their SO_2_ emissions reduction measures, such as desulfurization facility construction in Yunnan [41] and increasing structural emission reduction intensity and eliminating 250,000 kilowatts of power generation capacity in Anhui in 2010 [42], have not offset the role of economic growth in promoting the increase in SO_2_ emissions. This may be owing to the “strategy for the development of the western region” and the “rise of the central region” ideals, which have accelerated the modernization of inland areas [43]. Moreover, the developed coastal provinces and some underdeveloped provinces will transfer SO_2_ emissions to the central and western provinces with abundant energy resources and low productivity through industrial transfer and intermediate product trade [29].

### 4.2. Spatial Autocorrelation Analysis

Moran’s *I* has been used to study the spatial concentration of pollutant emissions [14]; for example, a study of industrial sewage at different scales in China found that the degree of concentration of prefectural-level industrial wastewater discharge was higher than that of provincial industrial wastewater [14], while another study analyzed Moran’s *I* of CO_2_ emission intensity in China from 1991 to 2010 and found that there was a significant aggregation in both high- and low-discharge provinces [15]. Additionally, Moran’s *I* has been used to study the degree of spatial agglomeration of PM_2.5_ [16]. Our Moran’s *I* results showed that, during the study period, there was a strong positive spatial auto-correlation between neighboring provinces in the YREB, with a trend of first increasing and then decreasing (Table 2), showing that the SO_2_ emissions in the YREB have significant agglomeration, and the emission intensity of one province affects that of the neigh-boring provinces. This effect is caused by the positive guidance by high-emission provinces of their neighboring provinces, promoting their learning and introducing them to new technologies to improve SO_2_ emission efficiency [44]. Therefore, when conducting emission reduction work, the SO_2_ emission accumulation phenomenon should be broken. Specifically, provinces should strengthen cooperation with other regions, realize cross-regional joint environmental governance, and establish a mechanism for coordination and co-governance of local governments [44]. The Chinese government should also promote the sharing and exchange of information and technology among provinces and formulate corresponding policies to strengthen development among provinces [15]. For example, the Yangtze River Delta region has set up a cooperation group for air pollution prevention and control, established and constantly improved a regional cooperation mechanism for pollution prevention and control, implemented multi-field information sharing, and carried out cross-regional and multi-department law enforcement linkage to win the blue sky battle [45].

### 4.3. Environmental Kuznets Curve

The relationship between environmental pollution and economic level is usually expressed by the EKC [46]. The EKC assumes that environmental quality is initially degraded during the improvement of per capita income level, but gradually improves when the income reaches the inflection point, that is, the relationship between environmental quality and income is an inverted U-shaped [47]. Previous studies have shown that the EKC shows other trends, including type U, N, inverted N, monotonous decreasing, and monotonous increasing trends [14,18]. We found that the EKC of SO_2_ emissions and per capita GDP in the YREB is N-shaped (Figure 3a) and that the 11 provinces in the YREB show N-shaped and inverted U-shaped curves (Figure 3b,c). Among them, the inverted U-shape indicates that as the economy grows, SO_2_ emissions eventually show a trend of rising and then falling. Overall, SO_2_ emissions in the YREB have slowed down and the pollution caused by SO_2_ emissions in these provinces (Jiangsu, Yunnan, and Hubei) has improved with regional economic development. For these regions, the political measures that the Chinese government was committed to promoting, such as technological innovation, market-oriented reform, and environmental regulations [48], have achieved good results. The progress of green technology and the improvement of production efficiency promoted by economic development [49] can also greatly reduce SO_2_ emissions. Therefore, as long as these provinces continue to maintain SO_2_ emission management policies and measures and actively create and introduce new technologies in future development, they can achieve SO_2_ emission reduction. However, the overall EKC of the YREB is N-shaped, indicating that with economic development, SO_2_ emission pollution first deteriorates, then improves, and finally deteriorates again. Notably, the EKCs of most YREB provinces are N-shaped. This suggests that the Chinese government issuing a series of SO_2_ emission reduction policies, such as those during the “11th five-year plan” period; shutting down backward technology to reduce the high pollution emissions of small, aging production facilities; and asking new factories to adopt advanced production technology, strictly abide by the emission standards, etc. played a positive role, allowing all provinces along the YREB to reach the EKC turning point [50]. However, in provinces with N-shaped EKCs, SO_2_ pollution will still increase with increasing economic level, indicating that economic development cannot ultimately solve the problem of SO_2_ pollution [10]. This may be because, as environmental protections in a province are strengthened, pollutants will migrate to areas with weak environmental protections, thus increasing the outflow of labor and capital in the province [19], leading to a decline in its environmental protection level. Economic development leads to an increase of industrial activities, resulting in an increase in production pollutants; however, research into innovative and environmentally friendly technologies to address these pollutants may be too costly to continue [19]. Therefore, these regions can increase green research and development investments [27], develop the use of renewable energy to optimize the energy structure [26], and improve energy efficiency by improving the quality of fossil fuels and the technological level of the energy industry [3]. To cope with SO_2_ emission increases caused by the increase of industrial activities with economic development, the government can raise pollution tax and formulate a tax rate that reflects the emission reduction costs [8]. Additionally, enterprises can be encouraged to upgrade production technology by means of financial subsidies [9]. Through the above analysis, it can be seen that with economic development, the EKC of the YREB and its provinces reached the first inflection point, showing an inverted U-shape. This is because, in the early stage of development, people pay more attention to economic development than to environmental protection, technology is used to develop resources, and residents lack the economic resources to pay for emission reduction, thus leading to a large amount of SO_2_ emissions in the early stage [43]. However, in the later stage of industrialization, with the development of economy and technology, people began to pay attention to environmental pollution, formulate strict environmental laws and regulations to prevent environmental destruction, and use technology and resources to reduce the emission of SO_2_, thus reaching the first inflection point [43]. However, as the economy continues to develop, some provinces reach a second inflection point, forming an N-shaped curve. This is because as the economy grows, more pollutants are produced, and the costs related to environmental protection rise [13]. In addition, highly polluting enterprises in economically developed areas may migrate to nearby underdeveloped areas with weak environmental protection systems, which will lead to capital loss in economically developed areas, affect their environmental protection fund investment, and ultimately cause environmental degradation [13].

### 4.4. Influence of Driving Factors of SO_2_ Emissions

The LMDI is often used to analyze the driving factors of pollutant emissions [25,26]. For example, CO_2_ [51], NO_X_ [52], industrial wastewater [14,22], PM_2.5_ [53], and SO_2_ [26]. One study found that energy consumption increases were the dominant reason for the increases of SO_2_ emissions, while technological effect was the dominant factor in SO_2_ emission reduction [26]. Our research results also showed that technological effect (approximately −1387.97 × 10^4^–130.24 × 10^4^ t) was the primary factor for reducing SO_2_ emissions in the YREB (Table 3). After 2006, China started to build new flue gas desulfurization equipment and the existing coal-fired power plants installed flue gas desulfurization equipment [28], the proportion of desulfurization in the iron and steel industries increased from 0 to 15.6% in 2010 [50]. The SO_2_ emissions in the YREB also began to decline in 2006 (Figure 2a); however, our study further found that the contribution value of the technology effect eventually decreased (Table 3), potentially owing to the hysteresis of the technological effect [54] and the high cost of technological research, which leads to the speed of technological progress being unable to catch up to the increase of SO_2_ production with economic growth. Additionally, technology has a double-sided effect. Technological progress can reduce pollution but may also bring new pollution risks [54]. It is crucial for governments at all levels to assign importance to technology. Therefore, in the future development of the YREB, investment in scientific and technological research and development can be increased to improve the innovation level of enterprises to promote the technological improvements [9]. Meanwhile, governments can also encourage enterprises to adopt advanced green and energy-efficient technologies, such as biological desulfurization technologies [55]. 

The emission reduction of SO_2_ should not rely solely on technological improvements, but should consider SO_2_ emission reduction at the source. For example, energy consumption reduction, renewable energy development [26], and energy utilization rate improvements. In this study, economic growth was the most important cause of SO_2_ emission increases because economic development has brought about a significant increase in personal income, which promotes a greater demand for energy products [53], leading to an increase in energy consumption [56]. However, economic growth will increase investment in green technological innovation [53], improve technological level, and reduce SO_2_ emissions. Previous studies have found that economic development cannot dissolve SO_2_ pollution on its own [10]. Additionally, although the economic effect on the SO_2_ emissions of each province eventually showed a downward trend, its positive contribution value was still the largest in our study (Table S2). Therefore, to reduce SO_2_ emissions, future development in the YREB needs to accelerate the transformation of the economic development mode from a high-speed growth to a high-quality development mode [53] and adjust the energy consumption structure to improve energy utilization efficiency [56]. We also found that the industrial structure effect played a significant role in SO_2_ emissions. During the study period, the effect of industrial structure inhibited SO_2_ emissions in Shanghai and Yunnan, but promoted SO_2_ emissions in the remaining nine provinces (Table S2). However, this showed that the adjustment of the industrial structure in these provinces has great potential for SO_2_ emission reduction. Therefore, these provinces can achieve SO_2_ emission reduction goals by optimizing industrial structures. Specifically, the government can encourage non-industrial enterprises to drive economic development, reduce the proportion of secondary industry, and increase the proportion of primary and tertiary industry enterprises in the industrial structure [57]. 

We also found that the population effect had the smallest impact on SO_2_ emissions among the four driving factors (Table S2) and had different effects on the different provinces. For most provinces, the population effect ultimately showed a positive effect on SO_2_ emissions; however, it had a negative impact on Sichuan and Guizhou (Table S2). This may be because although the increase of population produced environmental pressures, it also improved the efficiency of urban infrastructure [56]. Citizens use public opinion to cause government pressure and attention to the environment, thus promoting the formation of informal environmental regulations, improving energy efficiency, and ultimately reducing SO_2_ emissions [56]. The government can increase urban infrastructure, reduce the sharing of polluting facilities, form informal environmental regulations [56], improve residential awareness of environmental protection, and advocate green and low-carbon lifestyles [9] to eliminate the positive impact of population effect on SO_2_ emissions.

### 4.5. Limitations

There are two limitations to this study. First, this study only discussed the spatiotemporal characteristics and influencing factors of SO_2_ emissions in the YREB at the provincial scale but ignored the differences between different cities within a province and the different effects of the same driving factors at different scales. Second, there is no data collected after 2017, thus restricting the study period to 2017. In the five years since, the spatiotemporal characteristics of SO_2_ emissions and the role of its driving factors may have changed. Therefore, on the premise of available data, future studies can use the latest SO_2_ emission data to analyze the spatiotemporal characteristics and influencing factors of SO_2_ emissions in the YREB at different scales.

## 5. Conclusions

In this study, we used spatial autocorrelation and the EKC and LMDI models to analyze the spatiotemporal characteristics and driving factors of SO_2_ emissions in the 11 provinces of the YREB from 1997 to 2017. Our results show that the total SO_2_ emissions in the YREB increased from 513.14 × 10^4^ t in 1997 to 974 × 10^4^ t in 2006 and then decreased to 321.9 × 10^4^ t in 2017. SO_2_ emissions increased before decreasing in 11 provinces, but the turning points were different. For example, emissions began decreasing in 2011 and 2015 in Yunnan and Anhui, respectively, while emissions in the remaining nine provinces began decreasing in 2005–2006. Additionally, the SO_2_ emissions of the YREB show a significant agglomeration phenomenon, and Moran’s *I* increased from 0.308 to 0.987 and then decreased to 0.233. Furthermore, the EKC of SO_2_ emissions and per capita GDP in the YREB is N-shaped, and the EKCs of the 11 provinces were either N-shaped (Shanghai, Zhejiang, Anhui, Jiangxi, Sichuan, Guizhou, Hunan, and Chongqing) or inverted U-shaped (Jiangsu, Yunnan, and Hubei). Thus, economic development can both promote and inhibit the emission of SO_2_. Finally, during the study period, the technical effect contributed the most (approximately −1387.97 × 10^4^–130.24 × 10^4^ t), followed by the economic effect (approximately 27.8×10^4^–1255.59 × 10^4^ t), the structural effect (approximately −56.45 × 10^4^–343.90 × 10^4^ t), and finally, the population effect (approximately 4.25 × 10^4^–39.70 × 10^4^ t). Among these, the technical effect contributed most significantly to SO_2_ emission reduction, and the economic effect contributed most significantly to SO_2_ emission increases. Our results show that in 1997–2017 overall YREB SO_2_ emission reduction was most strongly promoted by science and technology, and local governments should establish collaboration and work mechanisms, promote information and technology sharing and communication between provinces, increase technological research and development investments, and encourage enterprises to use green and advanced energy saving technology.

## Figures and Tables

**Table 1:** SO_2_ emission driving factors and previous research methods.

Authors	Research Areas	Study Time Ranges (Year)	Methods	Driving Factors
[3]	China	2001–2007	The STIRPAT model	Energy investment and economic performance
[10]	China’s 29 provinces	2002–2015	MRIO-SDA approach	Economic growth and energy efficiency
[12]	26 cities of Yangtze River Delta	2005–2018	Moran’s Index, spatial econometrics model	Foreign direct investment, industrial structure, research and development investment, population size, energy intensity
[13]	139 Indian cities	2001–2013	Environmental Kuznets Curve	Economic growth
[23]	China’s 30 provinces	2004–2014	Panel data model, coefficient of divergence, STIRPAT model	Economic scale, technological progress, total population
[24]	China	1997–2012	Structural decomposition analysis	China’s demand structure
[26]	China	1995–2014	Logarithmic mean Divisia index	Technological progress, energy structure, energy consumption

**Table 2:** Global Moran’s *I* of SO_2_ emission changes in Yangtze River Economic Belt in 1997–2017, China.

Year	Moran’s *I*	*z*-Value	E(*I*)	SD	*P*	Mean
1997	0.308	4.530	−0.100	0.089	0.001	−0.096
1998	0.426	4.553	−0.100	0.116	0.001	−0.102
1999	0.720	4.510	−0.100	0.182	0.001	−0.102
2000	0.946	4.170	−0.100	0.251	0.001	−0.099
2001	0.940	4.437	−0.100	0.233	0.001	−0.095
2002	0.938	4.402	−0.100	0.235	0.001	−0.095
2003	0.966	4.115	−0.100	0.260	0.001	−0.103
2004	0.979	4.100	−0.100	0.262	0.001	−0.097
2005	0.987	4.159	−0.100	0.262	0.001	−0.104
2006	0.921	3.988	−0.100	0.254	0.001	−0.093
2007	0.905	4.046	−0.100	0.248	0.001	−0.096
2008	0.942	3.353	−0.100	0.311	0.001	−0.101
2009	0.914	4.050	−0.100	0.251	0.001	−0.102
2010	0.886	4.254	−0.100	0.232	0.001	−0.102
2011	0.597	4.317	−0.100	0.161	0.002	−0.097
2012	0.584	4.255	−0.100	0.160	0.002	−0.096
2013	0.484	4.160	−0.100	0.140	0.002	−0.098
2014	0.339	3.673	−0.100	0.119	0.001	−0.097
2015	0.233	3.188	−0.100	0.103	0.004	−0.095
2016	0.669	4.361	−0.100	0.175	0.001	−0.096
2017	0.265	3.887	−0.100	0.092	0.001	−0.094

E (*I*) is the value of mathematical expectation, SD is the standard deviation, *P*(*I*) is the significance level, *Z* represents the correlation between industrial wastewater and its location, and *I* is the Moran index.

**Table 3:** Decomposition analysis results of SO_2_ emission changes in Yangtze River Economic Belt in 1997–2017, China (unit: 10^4^ t).

	∆W_tec_	∆W_str_	∆W_eco_	∆W_pop_
1998	130.24	−56.45	27.81	4.25
1999	32.21	−31.22	58.51	7.91
2000	24.06	−21.11	118.47	8.21
2001	−90.12	1.51	158.71	16.59
2002	−182.43	32.09	215.00	19.39
2003	−142.97	101.07	352.69	28.35
2004	−296.23	170.99	474.67	32.93
2005	−415.76	230.13	618.25	12.64
2006	−572.17	285.48	731.74	15.81
2007	−767.79	307.41	851.70	18.53
2008	−946.23	342.93	931.76	21.71
2009	−1011.43	325.81	974.45	24.73
2010	−1154.11	343.90	1078.82	29.24
2011	−1274.68	340.41	1160.52	31.11
2012	−1323.21	317.42	1193.84	33.13
2013	−1366.91	294.51	1229.86	35.68
2014	−1387.97	260.35	1255.59	37.63
2015	−1386.69	214.06	1254.68	39.70
2016	−1343.98	160.07	1041.50	34.62
2017	−1330.47	129.03	976.72	33.56

∆W_tec_ represents the contribution of science and technology to SO_2_ emission, ∆W_str_ represents the contribution value of industrial structure to SO_2_ emission, ∆W_eco_ represents the contribution value of economic development to SO_2_ emission, ∆W_pop_ represents the contribution value of the total population to SO_2_ emission.

## Data Availability

The datasets used and/or analyzed during the current study are available from the author upon reasonable request—Zhen’an Yang (yza2765@126.com).

## Supplementary Materials

The following are available online at www.mdpi.com/article/10.3390/ijerph191710770/s1. Table S1: Fitting results of Environmental Kuznets Curve in the Yangtze River Economic Belt and different provinces. Table S2: Decomposition analysis results of SO_2_ emission changes of 11 provinces in the Yangtze River Economic Belt in 1997–2017, China (unit: 10^4^ t). Figure S1: Average SO_2_ emissions in the Yangtze River Economic Belt and its different provinces during 1997–2017.

## References

[1] Ren Y.A., Shen G.F., Shen H.Z., Zhong Q.R., Xu H.R., Meng W.J., Zhang W.X., Yu X.Y., Yun X., Luo Z.H. (2021). Contributions of biomass burning to global and regional SO_2_ emissions. Atmos. Res..

[2] Chen R., Zhang T., Guo Y., Wang J., Wei J., Yu Q. (2021). Recent advances in simultaneous removal of SO_2_ and NOx from exhaust gases: Removal process, mechanism and kinetics. Chem. Eng. J..

[3] Ahmad M., Zhao Z.Y., Irfan M., Mukeshimana M.C., Rehman A., Jabeen G., Li H. (2020). Modeling heterogeneous dynamic interactions among energy investment, SO_2_ emissions and economic performance in regional China. Environ. Sci. Pollut. Res..

[4] Xu X., Ding H., Wang X. (1995). Acute effects of total suspended particles and sulfur dioxides on preterm delivery: A community-based cohort study. Arch. Environ. Health.

[5] Khaniabadi Y.O., Daryanoosh S.M., Hopke P.K., Ferrante M., Marco A.D., Sicard P., Conti G.O., Goudarzi G., Basiri H., Mohammadi M.J. (2017). Acute myocardial infarction and COPD attributed to ambient SO_2_ in Iran. Environ. Res..

[6] Ghozikali M.G., Mosaferi M., Safari G.H., Jaafari J. (2015). Effect of exposure to O_3_, NO_2_, and SO_2_ on chronic obstructive pulmonary disease hospitalizations in Tabriz, Iran. Environ. Sci. Pollut. Res..

[7] Nascimento A.P., Santos J.M., Mill J.G., Albuquerque T.T.d.A.A., Júnior N.C.R., Reisen V.A., Pagel É.C. (2020). Association between the incidence of acute respiratory diseases in children and ambient concentrations of SO_2_, PM_10_ and chemical elements in fine particles. Environ. Res..

[8] Xie H.M., Shen M.H., Wei C. (2016). Technical efficiency, shadow price and substitutability of Chinese industrial SO_2_ emissions: A parametric approach. J. Clean Prod..

[9] Jiang L., He S., Cui Y., Zhou H., Kong H. (2020). Effects of the socio-economic influencing factors on SO_2_ pollution in Chinese cities: A spatial econometric analysis based on satellite observed data. J. Environ. Manag..

[10] Hu B., Li Z.T., Zhang L. (2019). Long-run dynamics of sulphur dioxide emissions, economic growth, and energy efficiency in China. J. Clean Prod..

[11] Liu Y., Wang S.J., Qiao Z.X., Wang Y.H., Ding Y.Y., Miao C.H. (2019). Estimating the dynamic effects of socioeconomic development on industrial SO_2_ emissions in Chinese cities using a DPSIR causal framework. Resour. Conserv. Recycl..

[12] Guo Z., Chen S.S., Yao S., Mkumbo A.C. (2021). Does foreign direct investment affect SO_2_ emissions in the Yangtze River Delta? A spatial econometric analysis. Chin. Geogr. Sci..

[13] Sinha A., Bhattacharya J. (2017). Estimation of environmental Kuznets curve for SO_2_ emission: A case of Indian cities. Ecol. Indic..

[14] Ma B.R., Tian G.J., Kong L.Q. (2020). Spatial-temporal characteristics of China’s industrial wastewater discharge at different scales. Environ. Sci. Pollut. Res..

[15] Zhao X., Burnett J.W., Fletcher J.J. (2014). Spatial analysis of China province-level CO_2_ emission intensity. Renew. Sustain. Energy Rev..

[16] Wu Q.L., Guo R.X., Luo J.H., Chen C. (2021). Spatiotemporal evolution and the driving factors of PM_2.5_ in Chinese urban agglomerations between 2000 and 2017. Ecol. Indic..

[17] Jiang Q.Q., Khattak S.I., Rahman Z.U. (2021). Measuring the simultaneous effects of electricity consumption and production on carbon dioxide emissions (CO_2_e) in China: New evidence from an EKC-based assessment. Energy.

[18] Han C.Y., Gu Z.L., Yang H.X. (2021). EKC Test of the relationship between nitrogen dioxide pollution and economic growth-A spatial econometric analysis based on Chinese City Data. Int. J. Environ. Res. Public Health.

[19] Moutinho V., Varum C., Madaleno M. (2017). How economic growth affects emissions? An investigation of the environmental Kuznets curve in Portuguese and Spanish economic activity sectors. Energy Policy.

[20] Hille E., Lambernd B., Tiwari A.K. (2021). Any signs of green growth? A spatial panel analysis of regional air pollution in South Korea. Environ. Resour. Econ..

[21] Wang K.F., Zhu Y.L., Zhang J.P. (2021). Decoupling economic development from municipal solid waste generation in China’s cities: Assessment and prediction based on Tapio method and EKC models. Waste Manag..

[22] Guo H., Zhang Y.W., Yang Z.A. (2022). Quantification of industrial wastewater discharge from the major cities in Sichuan province, China. Environ. Sci. Pollut. Res..

[23] Zhao D.T., Chen H., Li X.D., Ma X.T. (2018). Air pollution and its influential factors in China’s hot spots. J. Clean Prod..

[24] Jiao J.L., Han X.F., Li F.Y., Bai Y., Yu Y.D. (2017). Contribution of demand shifts to industrial SO_2_ emissions in a transition economy: Evidence from China. J. Clean Prod..

[25] Xing Z.C., Wang J.G., Feng K.S., Hubacek K. (2020). Decline of net SO_2_ emission intensity in China’s thermal power generation: Decomposition and attribution analysis. Sci. Total Environ..

[26] Yang X., Wang S.J., Zhang W.Z., Li J.M., Zou Y.F. (2016). Impacts of energy consumption, energy structure, and treatment technology on SO_2_ emissions: A multi-scale LMDI decomposition analysis in China. Appl. Energy.

[27] Tang Y., Chen S.X., Huang J.B. (2021). Green research and development activities and SO_2_ intensity: An analysis for China. Environ. Sci. Pollut. Res..

[28] Cao J., Garbaccio R., Ho M.S. (2009). China’s 11th Five-Year Plan and the environment: Reducing SO_2_ emissions. Rev. Environ. Econ. Policy.

[29] Chen X.Q., Liu W., Zhang J.Q., Li Z.P. (2020). The change pattern and driving factors of embodied SO_2_ emissions in China’s inter-provincial trade. J. Clean Prod..

[30] Zhou K., Wu J.X., Liu H.C. (2021). Spatiotemporal variations and determinants of water pollutant discharge in the Yangtze River Economic Belt, China: A spatial econometric analysis. Environ. Pollut..

[31] Wang K.L., Xu R.Y., Zhang F.Q., Miao Z., Peng G. (2021). Spatiotemporal heterogeneity and driving factors of PM_2.5_ reduction efficiency: An empirical analysis of three urban agglomerations in the Yangtze River Economic Belt, China. Ecol. Indic..

[32] Research Center for Yangtze River Delta and Yangtze River Economic Belt, 2020 Report on Social Development of the Yangtze River Economic Belt (2019–2020). https://cyrdebr.sass.org.cn/2020/1223/c5775a100922/page.htm.

[33] Yang Z., Zhang X.L., Lei J., Duan Z.L., Li J.G. (2019). Spatio-temporal pattern characteristics of relationship between urbanization and economic development at county level in China. Chin. Geogr. Sci..

[34] The Central People’s Government of the People’s Republic of China The Eleventh Five-Year Plan of National Environmental Protection. http://www.gov.cn/zwgk/2007-11/26/content_815498.htm.

[35] The Central People’s Government of the People’s Republic of China Action Plan of Air Pollution Prevention and Control. http://www.gov.cn/zwgk/2013-09/12/content_2486773.htm.

[36] The Central People’s Government of the People’s Republic of China The Twelfth Five-Year Plan for Energy Saving and Emission Reduction. http://www.gov.cn/zwgk/2012-08/21/content_2207867.htm.

[37] Jiangsu Provincial Department of Ecology and Environment Environmental Status Bulletin of Jiangsu Province in 2008. http://sthjt.jiangsu.gov.cn/art/2009/6/5/art_8385510135263.html.

[38] Sichuan Provincial Department of Ecology and Environment Environmental Status Bulletin of Sichuan Province in 2013. http://sthjt.sc.gov.cn/sthjt/c104157/hjglnew.shtml.

[39] National Bureau of Statistics of China (2003–2004) China Statistical Yearbook. http://www.stats.gov.cn/tjsj/ndsj/.

[40] Standing Committee of the National People’s Congress Air Pollution Prevention and Control Law of the People’s Republic of China. http://www.gov.cn/zhengce/2015-08/30/content_2922326.htm.

[41] Yunnan Provincial Department of Ecology and Environment Environmental Status Bulletin of Yunnan Province in 2005. http://sthjt.yn.gov.cn/hjzl/hjzkgb/200605/t20060530_11002.html.

[42] Anhui Provincial Department of Ecology and Environment Environmental Status Bulletin of Anhui Province in 2010. https://sthjt.ah.gov.cn/public/21691/112837691.html.

[43] Lu C.X., Venevsky S., Shi X.L., Wang L.Y., Wright J.S., Wu C. (2021). Econometrics of the environmental Kuznets curve: Testing advancement to carbon intensity-oriented sustainability for eight economic zones in China. J. Clean Prod..

[44] Zhang Y.J., Hao J.F., Song J. (2016). The CO_2_ emission efficiency, reduction potential and spatial clustering in China’s industry: Evidence from the regional level. Appl. Energy.

[45] Ministry of Ecology and Environment, PRC, 2020 The Yangtze River Delta Regional Pollution Prevention and Control Cooperative Mechanism Meeting Was Held. https://www.mee.gov.cn/ywdt/hjywnews/202006/t20200607_783124.shtml.

[46] Wu Q.L., Gu S.T. (2021). Discerning drivers and future reduction paths of energy-related CO_2_ emissions in China: Combining EKC with three-layer LMDI. Environ. Sci. Pollut. Res..

[47] Pérez-Suárez R., López-Menéndez A.J. (2015). Growing green? Forecasting CO_2_ emissions with Environmental Kuznets Curves and Logistic Growth Models. Environ. Sci. Policy.

[48] Zhou X., Yu Y., Yang F., Shi Q.F. (2021). Spatial-temporal heterogeneity of green innovation in China. J. Clean Prod..

[49] Balado-Naves R., Baños-Pino J.F., Mayor M. (2018). Do countries influence neighbouring pollution? A spatial analysis of the EKC for CO_2_ emissions. Energy Policy.

[50] Liu Q.L., Wang Q. (2017). How China achieved its 11th Five-Year Plan emissions reduction target: A structural decomposition analysis of industrial SO_2_ and chemical oxygen demand. Sci. Total Environ..

[51] Chong C.H., Tan W.X., Ting Z.J., Liu P., Ma L.W., Li Z., Ni W.D. (2019). The driving factors of energy-related CO_2_ emission growth in Malaysia: The LMDI decomposition method based on energy allocation analysis. Renew. Sustain. Energy Rev..

[52] Wang L.K., Wang Y., He H., Lu Y.L., Zhou Z.H. (2020). Driving force analysis of the nitrogen oxides intensity related to electricity sector in China based on the LMDI method. J. Clean Prod..

[53] Zhang Y., Shuai C.Y., Bian J., Chen X., Wu Y., Shen L.Y. (2019). Socioeconomic factors of PM_2.5_ concentrations in 152 Chinese cities: Decomposition analysis using LMDI. J. Clean Prod..

[54] Zhou Z.M., Ye X.Y., Ge X.Y. (2017). The impacts of technical progress on sulfur dioxide Kuznets Curve in China: A spatial panel data approach. Sustainability.

[55] Makgato S.S., Chirwa E.M.N. (2020). The desulphurization potential of waterberg steam coal using bacteria isolated from coal: The SO_2_ emissions control technique. J. Clean Prod..

[56] Liu Q.L., Long Y., Wang C.R., Wang Z., Wang Q., Guan D.B. (2019). Drivers of provincial SO_2_ emissions in China-Based on multi-regional input-output analysis. J. Clean Prod..

[57] Wang Y., Shi L., Chen D., Tan X. (2020). Spatial-temporal analysis and driving factors decomposition of (de)coupling condition of SO_2_ emissions in China. Int. J. Environ. Res. Public Health.

