# Supplementary material for: Quantitative Analysis of Sulfur Dioxide Emissions in the Yangtze River Economic Belt from 1997 to 2017, China
Source: Int J Environ Res Public Health. 2022 Aug 29;19(17):10770. doi: 10.3390/ijerph191710770 (PMC9518338; doi:10.3390/ijerph191710770)
Supplement: Supplementary file 1 [file ijerph-19-10770-s001.zip › ijerph-1881849-supplementary.pdf]

Table S1. Fitting results of Environmental Kuznets Curve in Yangtze River Economic Belt and different provinces during.

Area	EKC type	Equation	*P*-value	R^2
Yangtze River Economic Belt	N	$y = 1.11*10^{-11}x^3 - 1.61*10^{-6}x^2 + 0.06x + 299.06$	$<$ 0.001	0.776
Chongqing		$y = 1.31*10^{-12}x^3 - 1.62*10^{-7}x^2 + 4.89x + 38.47$	$<$ 0.001	0.644
Sichuan		$y = 6.39*10^{-12}x^3 - 5.76*10^{-7}x^2 + 0.013x + 35.90$	$<$ 0.001	0.716
Guizhou		$y = 1.42*10^{-11}x^3 - 9.64*10^{-7}x^2 + 0.017x + 44.17$	0.004	0.455
Hunan		$y = 1.99*10^{-12}x^3 - 2.36*10^{-7}x^2 + 0.007x + 33.45$	$<$ 0.001	0.736
Jiangxi		$y = 1.69*10^{-12}x^3 - 2.23*10^{-7}x^2 + 0.007x - 1.05$	$<$ 0.001	0.815
Shanghai		$y = 1.77*10^{-13}x^3 - 4.76*10^{-8}x^2 + 0.003x - 25.67$	$<$ 0.001	0.823
Zhejiang		$y = 2.17*10^{-13}x^3 - 5.65*10^{-8}x^2 + 0.003x + 23.52$	$<$ 0.001	0.833
Anhui		$y = 1.14*10^{-12}x^3 - 1.54*10^{-7}x^2 + 0.004x + 18.37$	$<$ 0.001	0.788
Jiangsu	Inverted U	$y = -1.48*10^{-8}x^2 + 0.001x + 102.17$	$<$ 0.001	0.830
Yunnan		$y = -1.25*10^{-7}x^2 + 0.005x + 11.16$	$<$ 0.001	0.810
Hubei		$y = -4.67*10^{-8}x^2 + 0.003x + 36.83$	$<$ 0.001	0.721

Table S2. Decomposition analysis results of SO_2 emission changes of 11 provinces in Yangtze River Economic Belt in 1997-2017, China (unit: 104t).

	Shanghai				Jiangsu				Zhejiang			
	ΔWtec	ΔWstr	ΔWeco	ΔWpop	ΔWtec	ΔWstr	ΔWeco	ΔWpop	ΔWtec	ΔWstr	ΔWeco	ΔWpop
1998	-5.35	-0.85	5.00	0.19	20.98	-7.49	7.74	0.52	12.74	2.76	4.20	0.24
1999	-18.04	0.94	7.67	0.41	-21.82	1.79	12.82	0.87	5.32	6.19	7.77	0.45
2000	-19.93	-0.51	7.99	5.03	-19.20	7.08	22.15	4.25	-8.73	10.10	11.16	2.56
2001	-27.82	2.15	12.02	3.56	-36.49	9.20	33.25	2.98	-18.01	13.25	17.36	1.89
2002	-28.55	1.24	15.76	3.95	-58.18	16.42	44.16	3.29	-27.71	18.68	25.01	2.32
2003	-31.77	6.71	23.16	6.83	-75.83	30.73	65.40	3.95	-33.37	24.31	38.41	3.00
2004	-38.69	7.58	30.61	7.80	-111.81	47.15	84.49	4.36	-46.83	33.69	49.78	3.67
2005	-45.10	10.53	36.73	9.05	-133.04	53.21	112.06	5.26	-53.84	35.96	56.76	6.04
2006	-52.53	11.27	42.03	9.94	-162.75	60.92	126.05	6.26	-66.97	39.25	65.49	7.04
2007	-58.64	8.15	49.22	10.87	-189.57	61.56	142.96	7.14	-82.22	39.77	73.38	7.68
2008	-62.23	4.77	51.00	10.96	-206.69	56.97	155.40	7.59	-100.61	48.33	77.25	8.04
2009	-61.07	-1.33	49.43	10.78	-215.85	53.49	161.90	8.04	-101.44	43.90	78.13	8.43
2010	-68.72	0.98	46.09	17.35	-231.87	50.32	176.89	9.84	-111.28	43.26	83.77	10.96
2011	-72.47	0.41	41.01	14.95	-246.77	48.27	193.81	10.25	-118.92	42.59	90.47	10.98
2012	-71.89	-1.99	41.57	15.04	-252.42	44.40	197.20	10.21	-120.49	39.65	91.55	10.78
2013	-72.35	-3.92	42.65	15.10	-257.70	40.25	201.62	10.18	-122.99	37.68	92.89	10.67
2014	-72.43	-5.60	42.40	14.34	-261.35	35.95	205.82	10.23	-123.65	34.82	94.58	10.57
2015	-71.32	-7.79	42.46	13.63	-262.08	29.37	206.38	10.02	-123.80	30.95	95.07	10.48
2016	-68.40	-6.61	32.50	9.83	-254.70	24.71	178.58	8.60	-113.80	21.88	69.92	7.74
2017	-62.48	-4.14	22.08	6.30	-249.47	21.79	161.24	7.69	-108.57	17.02	62.54	6.98

	Anhui				Jiangxi				Hubei				Hunan			
	ΔWtec	ΔWstr	ΔWeco	ΔWpop	ΔWtec	ΔWstr	ΔWeco	ΔWpop	ΔWtec	ΔWstr	ΔWeco	ΔWpop	ΔWtec	ΔWstr	ΔWeco	ΔWpop
1998	22.60	-18.48	0.22	0.33	7.34	-4.67	-0.16	0.23	14.97	-0.01	-4.73	0.25	29.60	-15.13	0.28	0.31
1999	17.09	-16.86	2.35	0.62	5.04	-4.78	1.32	0.44	13.93	0.70	-3.42	0.49	25.17	-15.18	3.26	0.55
2000	14.58	-18.12	6.17	-0.79	8.49	-5.22	3.95	-0.06	11.41	-0.49	0.05	1.16	20.09	-13.57	9.51	-0.21
2001	9.97	-17.57	8.14	1.10	2.35	-3.74	5.43	0.20	6.51	-1.85	4.35	0.75	8.71	-9.83	11.93	1.05
2002	3.90	-14.49	11.12	1.15	-2.48	-2.64	7.88	0.40	1.66	-1.61	7.72	0.83	-0.30	-5.96	15.72	1.30
2003	5.27	-11.69	16.91	1.76	7.89	-1.36	14.76	0.78	6.73	-0.28	14.66	1.06	9.75	-0.06	26.39	1.93
2004	0.18	-10.91	24.35	2.16	3.41	3.18	22.56	1.10	3.53	2.07	23.65	1.26	-7.65	8.09	37.74	2.30
2005	-6.98	-2.40	33.30	-0.05	-3.48	11.43	30.25	1.45	-4.44	3.89	35.17	-1.51	-25.01	19.60	51.94	-1.45
2006	-16.76	2.21	39.84	-0.12	-16.94	19.54	37.41	1.73	-11.07	5.32	44.89	-1.72	-40.89	26.12	62.64	-1.30
2007	-31.03	6.89	48.17	-0.06	-30.85	24.67	44.66	1.97	-31.01	9.52	55.10	-1.60	-62.98	31.56	76.13	-1.14
2008	-45.18	12.31	55.18	0.06	-47.52	32.48	49.52	2.16	-47.51	13.73	63.44	-1.44	-90.94	43.56	85.39	-0.84
2009	-52.29	12.99	59.95	0.03	-53.06	33.63	51.79	2.39	-57.57	15.57	68.94	-1.33	-98.81	42.53	91.12	-0.57
2010	-64.59	16.59	69.17	-1.19	-63.70	36.51	58.63	2.61	-70.89	18.12	78.64	-1.25	-115.56	46.61	101.23	1.00
2011	-75.93	19.12	77.65	-1.11	-72.35	38.36	67.85	2.90	-82.52	20.28	91.13	-1.02	-129.59	46.22	103.95	1.14
2012	-81.31	19.30	81.71	-0.96	-74.91	36.77	70.27	2.98	-89.39	19.87	93.87	-0.80	-135.34	44.95	106.60	1.47
2013	-85.62	18.57	84.60	-0.66	-78.35	35.99	73.38	3.10	-93.24	17.64	97.47	-0.62	-140.21	44.02	111.61	1.89
2014	-87.88	17.08	87.16	-0.29	-80.14	34.10	74.66	3.18	-95.25	14.33	101.08	-0.47	-143.61	42.17	114.75	2.23
2015	-86.89	13.75	87.81	0.11	-80.37	31.82	76.36	3.34	-97.28	12.79	101.11	-0.17	-143.58	36.95	116.82	2.54
2016	-85.16	10.03	69.72	0.34	-73.35	21.15	55.75	2.48	-94.65	8.83	75.63	0.07	-133.01	26.50	92.19	2.17
2017	-85.45	8.07	67.11	0.58	-71.60	18.22	50.96	2.33	-92.78	5.78	70.19	0.15	-124.03	19.83	76.91	1.93

	Chongqing				Sichuan				Guizhou				Yunan			
	ΔWtec	ΔWstr	ΔWeco	ΔWpop	ΔWtec	ΔWstr	ΔWeco	ΔWpop	ΔWtec	ΔWstr	ΔWeco	ΔWpop	ΔWtec	ΔWstr	ΔWeco	ΔWpop
1998	1.91	-12.17	6.76	0.22	14.69	-10.12	2.57	0.51	24.50	-12.00	10.69	1.04	2.10	-1.58	2.76	0.35
1999	36.45	-12.70	11.86	0.60	-9.39	-7.62	4.67	0.82	2.34	-11.49	14.27	1.83	0.60	-3.94	3.30	0.65
2000	18.18	-6.97	14.75	0.81	34.72	-12.97	14.51	-0.97	-7.51	-10.70	22.96	-1.42	2.99	-3.89	4.88	1.37
2001	5.42	-7.27	18.27	0.86	15.62	-5.75	18.17	1.92	-19.10	-10.64	22.88	3.08	-2.41	-2.88	6.39	1.30
2002	-3.46	-5.64	23.63	0.99	-1.86	3.35	25.20	2.21	-26.69	-8.11	27.90	3.68	-6.03	-1.87	8.56	1.60
2003	2.09	-2.24	35.52	1.60	5.37	8.56	39.78	2.82	12.35	-2.81	55.32	6.50	3.41	-1.86	14.30	2.36
2004	-10.57	3.54	45.38	1.49	-16.09	19.61	55.71	3.16	-9.83	5.63	67.49	7.28	-0.56	-2.21	20.78	2.75
2005	-15.44	4.19	60.22	-4.93	-44.95	38.73	74.54	-2.44	-34.09	17.66	88.14	3.16	-2.02	-1.70	25.68	3.19
2006	-35.01	17.83	68.31	-4.79	-69.48	46.61	89.76	-2.91	-51.94	27.12	106.40	4.00	-9.46	1.59	32.35	3.58
2007	-59.57	28.63	78.40	-4.52	-106.03	57.63	105.42	-3.23	-72.54	21.07	124.07	3.98	-18.64	1.26	39.85	3.78
2008	-82.74	37.56	87.64	-3.92	-124.45	58.01	120.19	-3.07	-107.31	30.95	134.45	4.48	-30.48	5.07	44.67	3.90
2009	-103.09	49.84	91.62	-3.43	-137.01	60.78	128.16	-2.55	-109.34	22.57	138.98	4.47	-31.16	2.31	47.60	4.11
2010	-115.88	51.28	99.81	-2.87	-160.27	67.16	146.12	-4.03	-126.20	25.08	158.15	-3.05	-39.34	4.49	53.53	4.37
2011	-125.22	46.24	100.01	-2.00	-177.90	62.83	144.67	-3.52	-142.36	22.03	173.05	-3.22	-39.00	2.32	73.23	5.53
2012	-127.38	41.88	103.85	-1.54	-185.04	59.79	150.77	-3.21	-158.09	22.10	181.95	-2.77	-45.87	1.79	78.55	5.70
2013	-129.23	38.22	107.24	-1.12	-190.50	57.32	153.56	-2.83	-172.79	23.37	189.43	-2.29	-50.00	-0.71	83.88	5.91
2014	-127.75	31.50	110.06	-0.78	-191.50	52.11	157.42	-2.51	-183.62	23.80	193.56	-2.08	-52.01	-3.10	85.71	6.03
2015	-129.63	29.57	110.35	-0.37	-183.77	41.24	152.01	-1.84	-185.65	16.64	194.93	-1.54	-52.52	-6.07	83.93	5.98
2016	-121.10	22.26	87.94	0.07	-173.69	29.26	130.26	-1.13	-185.47	14.54	175.61	-0.89	-53.97	-8.76	82.42	5.88
2017	-120.15	19.55	85.93	0.34	-169.95	21.84	123.67	-0.77	-196.20	14.59	189.90	-0.47	-57.30	-9.72	73.26	5.16

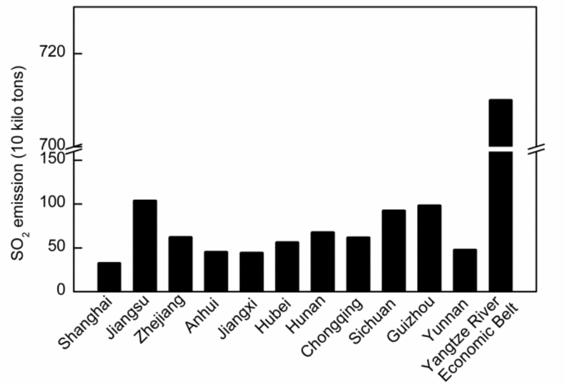

Figure S1. Average emission of SO_2 in Yangtze River Economic Belt and different provinces during 1997-2017.
